# Effect of maternal stress during pregnancy on the risk for preterm birth

**DOI:** 10.1186/s12884-015-0775-x

**Published:** 2016-01-15

**Authors:** Caroline Lilliecreutz, Johanna Larén, Gunilla Sydsjö, Ann Josefsson

**Affiliations:** Division of Obstetrics and Gynaecology, Department of Clinical and Experimental Medicine, Faculty of Health Sciences, Linköping University, SE-581 85 Linköping, Sweden

**Keywords:** Premature birth, Stress, Pregnancy, Attributable risk

## Abstract

**Background:**

Preterm birth defined as birth prior to 37 weeks of gestation is caused by different risk factors and implies an increased risk for disease and early death for the child. The aim of the study was to investigate the effect of maternal stress during pregnancy on the risk of preterm birth.

**Methods:**

A case–control study that included 340 women; 168 women who gave birth preterm and 172 women who gave birth at term. Data were manually extracted from standardized medical records. If the medical record contained a psychiatric diagnosis or a self-reported stressor e.g., depression or anxiety the woman was considered to have been exposed to stress during pregnancy.

Adjusted odds ratio (AOR) was used to calculate the attributable risk (AR) of maternal stress during pregnancy on preterm birth, both for the women exposed to stress during pregnancy (AR1 = (AOR-1)/AOR) and for the whole study population (AR2 = AR1*case fraction).

**Results:**

Maternal stress during pregnancy was more common among women who gave birth preterm compared to women who gave birth at term (*p* <0.000, AOR 2.15 (CI = 1.18–3.92)). Among the women who experienced stress during pregnancy 54 % gave birth preterm with stress as an attributable risk factor. Among all of the women the percentage was 23 %.

**Conclusions:**

Stress seems to increase the risk of preterm birth. It is of great importance to identify and possibly alleviate the exposure to stress during pregnancy and by doing so try to decrease the preterm birth rate.

## Background

Each year 18 000 children in the Nordic countries are born preterm i.e., 6.6 % of all births and 12.9 million worldwide i.e., 9.6 % [1]. Preterm birth is the major risk factor for perinatal mortality and morbidity and deaths among preterm infants represent 63 % of all deaths in children <5 years [1]. More than two-thirds of all perinatal deaths are associated with preterm birth [2]. Due to technological advances in neonatal care the survival rate for extreme preterm births has increased [3]. Preterm birth (<37 weeks of completed gestation) is associated with numerous risk factors and the causes is thought to be initiated by multiple mechanisms that might be interacting.

Multiple pregnancies, vaginal bleeding during pregnancy, and polyhydramnios or oligohydramnios are all associated with an increased risk for premature delivery [4]. Intrauterine infection leads to an activation of the immune system, and may subsequently lead to preterm birth. Low body mass index (BMI) during pregnancy is associated with increased risk for preterm delivery, and obesity is associated with disorders like preeclampsia and diabetes, which carry an increased risk for preterm birth. Maternal thyroid disease, asthma, hypertension as well as a history of cervical surgical procedures and uterus anomalies are associated with preterm birth [4]. Cigarette smoking carries an increased risk for preterm birth [5]. Nicotine and carbon monoxide are potent vasoconstrictors that are thought to cause placental damage and decreased uteroplacental blood flow [4]. Previous preterm delivery is also associated with an increased risk (OR 1.39, 95 % CI =1.29–1.50) for preterm birth in the next pregnancy [6]. These are all risk factors and probably indifferent of the presence of maternal stress.

Accumulating evidence both in human and animal studies suggests that maternal psychological and social stress during pregnancy represents conditions that may adversely affect the pregnancy outcome such as increased risk of morbidity for the child, lower birth weight and increase the risk for preterm birth. This is evident even after adjusting for other biomedical, socio-demographic and behavioral risk factors. The linking systems between mental and psychosocial processes within the mother to the fetus involve autonomic, neuroendocrine and immune systems [7–14]. Low socioeconomic and educational status, low and high maternal ages and single marital status have also been associated with preterm birth. Depression has been suggested to carry up to a 2-fold increased risk for preterm delivery [1]. In utero exposure to either continuous SSRI treatment for depression or continuous depression without treatment has been associated with preterm birth [15–17]. Experience of stress during pregnancy is important to consider when caring for the pregnant women. Preterm birth has an impact on the newborn baby, the family and the health care system. The quantity and the load of stress needed for stress to become a risk factor for adverse outcome is still unknown.

Hence our hypothesis was that maternal stress during pregnancy can attribute to the adverse outcome of preterm labor.

Thus, the aim of the study was to investigate the effect of maternal stress during pregnancy on the risk for preterm birth. This will be analyzed through the statistical method attributable risk (AR) which will be calculated in two different ways. First to estimate the proportion of women exposed to stress during pregnancy that give birth preterm due to stress as an attributable risk factor. Second to estimate the proportion of all women giving birth preterm due to stress during pregnancy as an attributable risk factor.

## Methods

The study was designed as a case–control study with prospectively collected data. All women who gave birth before gestational week 37 during one year (2010) at Linköping University hospital were identified with the ICD-code for premature labour O60 in the computerized chart system Obstetrix®. All pregnancies and deliveries are registered in the chart system along with medical information and diagnosis during the antenatal period and admission to the delivery ward. The medical information of all study subjects were retrieved by manually inspecting the entries in Obstetrix® and transferring information to a spreadsheet. This was done by a senior medical student together with the first author (C L) in 2011. One hundred and sixty-eight women were diagnosed with preterm delivery and constitute the women from the index group. The control group consists of 172 women who gave birth after gestational week 37 at the same hospital during the same year. They were consecutively chosen from the chart system and matched for parity and age +/− 5 years. As for the study subjects who had delivered preterm, these individuals were retrieved by manually checking the entries in Obstetrix®.

The total study population comprised 332 women as eight women in the index group experienced an intrauterine fetal death (IUFD) before term and therefore were excluded.

Risk factors for preterm birth were scrutinized in each woman’s medical record. Age, marital status (married/cohabiting or single), sick leave at first visit with the midwife (yes or no), level of socioeconomics/education (higher education, lower education, unemployed/parental leave, student), BMI in the beginning of the pregnancy (−24.9, 25–29.9, 30-), use of tobacco products (yes or no), alcohol or drugs (yes or no), and parity (0,1, 2 or more previous deliveries). Earlier pregnancies were divided into groups with no previous preterm deliveries and one or more previous preterm delivery, previous and/or current diseases (yes or no) e.g., diabetes, asthma, uterus malformation, cervix insufficiency, conization, systemic lupus erythematosus, fibromyalgia, urinary tract infections/bacteriuria, pyelonephritis, genital infection, inflammatory bowel disease, hepatitis, hypothyroidism, hyperthyroidism, mediterranean fever, and congenital heart defects. SSRI use during pregnancy (yes or no), twin pregnancies (yes or no), amniotic fluid pathology examined with ultrasonography (divided into three groups: normal amount of amniotic fluid, polyhydramnios and oligohydramnios) clinical signs of chorioamnionitis (yes or no), reported or observed premature contractions (yes or no), observed cervical changes (yes or no), reported or observed vaginal bleeding (yes or no), anemia defined as Hemoglobin <100 (yes or no), observed preterm premature rupture of membranes (PPROM) (yes or no), small for gestational age (SGA) defined as a birth weight < −2 SD of the mean weight for the gestational length, observed congenital defects by a pediatrician (yes or no), observed ablation (yes or no), observed preeclampsia (yes or no), onset of labor (spontaneous or induced), mode of delivery (vaginal, vaccum extraction or cesarean section), and gestational week at birth were manually extracted from the records.

*Maternal stress exposure was define as:* having a psychiatric diagnosis according to medical records (depression, anxiety, fear of childbirth, psychosis, phobia and/or eating disorder) or if the patient’s medical record revealed self-reported stress. Self-reported maternal stress could be due to the pregnancy itself, previous traumatic experiences from pregnancy and childbirth, fear of congenital defects in the unborn child or injury. Lack of social support or a problematic relationship between the woman and her partner or with significant others as well as economic and work related problems were considered as stress exposure. These women were considered to have been exposed to stress during pregnancy.

### Statistics

The statistical analyses were done with SPSS (version 18). The rejection of the null-hypothesis was set to 0.05 (two-sided) in all statistical analyses. Student t-test was used to test differences between quantitative variables. Pearson’s Chi-square test was used to for testing differences in frequencies between categories. The possible effects of confounders were estimated through multiple logistic regression analyses and the adjusted odds ratios (AOR) were presented with a 95 % confidence interval. In these models, preterm delivery was set as dependent variable and identified confounding factors were considered as independent variables. A variable theoretically causing both prematurity and maternal stress was considered a confounder. Of all the risk factors that were examined in this study; premature contractions, tobacco use, previous premature delivery, genital tract infection and twin pregnancy were considered as confounders. To verify these confounders correlations were computed. The correlations between stress and confounding factors were found to range between −0.062 and 0.268. Even though the correlations between stress and previous preterm delivery (ρ = 0.072), infection (ρ = 0.092), and twin pregnancy (ρ = −0.062) were low it was decided to keep this factors in the models based on previous theories on their importance with respect to preterm delivery [4].

Attributable risk (AR), was estimated in two different ways. AR1 = (AOR-1)/AOR, was used to estimate how many of the women exposed to stress during pregnancy had delivered preterm because of the exposure as an attributable risk factor. AR2 = AR1*case fraction, with case fraction standing for the proportion of women delivering preterm who were exposed to stress during pregnancy. AR2 estimates how many women who delivered preterm in our study population did so because of the exposure to stress during pregnancy as an attributable risk factor.

The present study was approved by the Ethical Review Board in Linköping, nr. 2011/183-31. Written informed consent for use of patient records in research is not required by the ethics committee standards.

## Results

The distribution of the socio-demographic variables is shown in Table 1. Tobacco use as well as sick leave in early pregnancy were more common among the women from the index group compared with the control group (p = 0.007; p = 0.014). There were no women with drug addiction and one woman had an alcohol addiction during pregnancy in the index group.

**Table 1:** Socio-demographic data for the study population

	Index group		Control group		
	*N*	%	*N*	%	*P*-value
					Index vs. Control
Age (mean/SD)**	30.6/6.09		30.3/5.16		0.602
Marital status*					0.989
Married/Cohabiting	133	92.4	150	93.2	
Single	11	5.6	11	6.8	
Sick leave at registration*					0.014
Yes	9	7.0	2	1.3	
No	120	93.0	156	98.7	
Socioeconomic group*					0.132
Higher education	41	33.6	63	40.1	
Lower education	47	38.5	52	33.1	
Unemployed/parental leave	21	17.2	16	10.2	
Student	13	10.7	26	16.6	
BMI in the beginning of pregnancy*					0.540
−24.9	60	45.8	80	50.6	
25–29.9	46	35.1	55	34.8	
30-	25	19.1	23	14.5	
Tobacco use*					0.007
Yes	30	22.6	17	10.7	
No	103	77.4	142	89.3	
Parity*					0.085
0	78	49.1	84	50.3	
1	43	27.0	58	34.7	
2 or more	38	23.9	25	15.0	

*P-value for Students t-test

**P-value for Pearson’s *x* ^2^-test

Previous and/or current diseases and conditions are shown in Table 2. Previous preterm birth was more common in the women from the index group compared with the control group (*p* <0.000). There were two women in the index group and one woman in the control group with a uterus malformation. One woman in the index group had a cervical insufficiency.

**Table 2:** Previous and/or current diseases and conditions

	Index group		Control group		
	*N*	%	*N*	%	*P*-value**
					Index vs. Control
Chronic disease/condition					0.882
Yes	27	16.9	27	15.7	
No	133	83.1	145	84.3	
Conization					0.308
Yes	10	6.3	6	3.5	
No	150	93.8	166	96.5	
Previous psychiatric diagnosis					0.416
Yes	18	11.3	25	14.5	
No	142	88.8	147	85.5	
Previous preterm delivery					<0.000
Yes	25	15.9	4	2.4	
No	132	84.1	163	97.6	

*Fisher’s exact test

**P-value for Person’s *x*2-test

Obstetric variables are shown in Table 3. Women in the index group more often had a genital tract infection (*p* <0.000), premature contractions (*p* <0.000) or PPROM (*p* <0.000). SGA (*p* <0.000) was also more common in the women from the index group compared to women who gave birth at term. Preeclampsia was more frequent among the women in the index group (*p* <0.000). In 11 of the 14 cases with preeclampsia in the women from the index group, the labor was induced. Eight women with preeclampsia in the index group were delivered by cesarean section because of the preeclampsia. Labor was induced in three of the five cases with preeclampsia in the control group.

**Table 3:** Obstetric variables

	Index group		Control group		
	*N*	%	*N*	%	*P*-value**
SSRI use during pregnancy					0.183
Yes	10	7	5	3.1	
No	132	93	156	96.9	
Twin pregnancy					<0.000
Yes	24	15	0	0	
No	136	85	165	100	
Amniotic fluid pathology					0.034
Normal	132	91.7	157	98.1	
Polyhydramnios	5	3.5	1	0.6	
Oligohydramnios	7	4.9	2	1.3	
Genital tract infection					<0.000
Yes	36	24.8	13	8.1	
No	109	75.2	147	91.9	
Premature contractions					<0.000
Yes	60	40.3	24	15	
No	89	59.7	136	85	
Cervical changes					<0.000
Yes	21	14.2	4	2.5	
No	127	85.8	156	97.5	
Vaginal bleeding					<0.000
Yes	59	40	15	9	
No	89	60	144	91	
Anemia					0.601
Yes	8	6	7	4	
No	126	94	152	96	
PPROM					<0.000
Yes	94	59.5	1	0.6	
No	64	40.5	164	99.4	
SGA					<0.000
Yes	24	15.3	4	2.4	
No	133	84.7	165	97.6	
Congenital defects					0.279
Yes	9	5.7	5	3	
No	149	94.3	164	97	
Placental abruption					0.003
Yes	8	5	0	0	
No	151	95	168	100	
Preeclampsia					<0.000
Yes	14	8.8	5	3	
No	145	91.2	163	97	
Maternal stress during pregnancy					<0.000
Yes	58	42.3	32	20	
No	79	57.7	128	80	
Onset of labor					0.005
Spontaneous	123	76.9	150	88.8	
Induced	37	23.1	19	11.2	
Mode of delivery					<0.000
Vaginal	98	61.6	143	84.6	
Vacuum extraction	8	5	13	7.7	
Cesarean section	53	33.3	13	7.7	
	Mean	SD	Mean	SD	p*
Gestational week at birth	33.06	3.84	40.03	1.2	<0.000

*P-value for Students t-test

**P-value for Pearson’s *x* ^2^-test

Induced labor and cesarean section were more frequent in the women from the index group (p = 0.005; *p* <0.000). Women who gave birth at term were more often doing so spontaneously and by vaginal birth compared to the women from the index group (p = 0.005; *p* <0.000).

The differences in premature contractions, previous preterm delivery and genital tract infection between the women from the index group and the control group remained after adjustment for the identified confounders maternal stress during pregnancy, premature contractions, tobacco use, previous preterm delivery and genital tract infection (Table 4).

**Table 4:** Unadjusted and adjusted odds ratios for risk factors among women giving birth preterm compared to women giving birth at term

	Unadjusted OR			Adjusted OR****		
	OR	95 % CI	P***	OR	95 % CI	P***
Maternal stress during pregnancy	2.94	1.76–4.92	<0.000	2.15	1.18–3.92	0.012
Premature contractions	3.82	2.22–6.58	<0.000	2.73	1.48–5.03	0.001
Tobacco use	2.43	1.27–4.64	0.007	1.63	0.77–3.45	0.205
Previous preterm delivery	7.72	2.62–22.73	<0.000	6.15	1.95–19.44	0.002
Genital tract infection	3.74	1.89–7.38	<0.000	3.18	1.47–6.87	0.003

*OR*, odds ratio, *CI* confidence interval

***P-value for Wald test

****Adjusted for maternal stress during pregnancy, premature contractions, tobacco use, previous preterm delivery and genital tract infection

Maternal stress during pregnancy was more common among women who delivered preterm (*p* <0.000) compared to the control group and was still evident after adjusting for premature contractions, tobacco use, previous preterm delivery and genital tract infection (Table 4). The difference also remained in the multiple logistic regressions with the same adjustment after excluding the 24 cases of twin pregnancies from the total study population.

There were 94 cases of PPROM in the women from the index group, and one woman with PPROM in the control group. Due to this, we could not estimate odds ratio (OR) for PPROM, and we were therefore unable to include PPROM in the multiple logistic regression. When excluding all cases of PPROM the unadjusted OR for maternal stress was estimated to be 2.82 (p = 0.002), compared to unadjusted OR, without exclusion of PPROM, at 2.94 (*p* <0.000). PPROM is considered more as a link in the process preceding preterm birth, rather than as a confounder.

There were 24 cases of twin pregnancies (no other multiple pregnancies were found in the study population) in the women from the index group, and no cases of twin pregnancies in the control group. As we were unable to calculate OR for twin pregnancies these were not included in the multiple logistic regression. OR for maternal stress after adjustment was estimated to be 2.15 (p = 0.012). After excluding all the cases of twin pregnancies from the women from the index group, the difference between the index group and the control group remained, when AOR for maternal stress was estimated to 1.91 (p = 0.049).

Attributable risk (AR) was estimated in two different ways. AR1 was estimated to be 54 %, when using the adjusted OR. This means that 54 % of the women exposed to stress during pregnancy delivered preterm because of the exposure as an attributable risk. AR2 was estimated to be 23 %. This means that 23 % of all the women who delivered preterm in our study population did so because of exposure to stress during their pregnancy as an attributable risk.

## Discussion

Several studies have previously suggested that women who experience psychological or social stress during pregnancy are at significantly increased risk for shorter gestation, earlier onset of spontaneous labor, low birthweight infant (<2500 g) and preterm delivery [7–15].

We have shown that maternal stress during pregnancy is more than twice as common among women who gave birth preterm compared to women who gave birth at term. The vast majority of the women exposed to stress during pregnancy, no matter what the origin or level of the stress was, gave birth preterm because of the exposure of stress as an attributable factor and in the total study population more than one fifth of the women gave birth preterm due to maternal stress during pregnancy as an attributable factor. Our study further establishes the already well-known risk factors like tobacco use, genital tract infection, previous preterm delivery and premature contractions.

The medical records in the Obstetrix® data system are standardized and prospectively recorded by midwives and obstetricians. The completion of information collected about women depends on accurate recording by medical staff and womens disclosure of personal information. This might be both strength and a limitation in the study. The effect is decreased or eliminated due to the fact that this problem exists in both the index and the control group.

Another limitation in this study is the awareness of the pregnancy outcome as the medical records are retrospectively scrutinized. There is a risk that a woman with preterm delivery will be classified as having been exposed to stress during the pregnancy because of preconceived ideas about stress. This weakness has perhaps been decreased through discussions with a senior obstetrician/researcher not aware of the delivery being preterm or full term. A strength in this study is that the material comes from an unselected group of women giving birth during 1 year.

In the study we have not divided the material into spontaneous or induced labor. The reason for that is multiple; medical treatment sometimes prevents spontaneous start, induction of labor could be due to conditions caused by for example stress or infection. An induction is sometimes started shortly before spontaneous labor. The medical practice is therefore something that interferes with the natural course.

In order to be considered a confounder in this study, the factor must be able to provoke both maternal stress and preterm birth independently. Thus sick leave in early pregnancy, amniotic fluid pathology, cervical changes, vaginal bleeding, placental abruption and preeclampsia where not considered as confounders and were therefore not adjusted for in the multiple logistic regression analyses. Twin pregnancies are associated with a high risk for preterm birth, with almost 60 % of all twins being delivered preterm. The cause is thought to be due to uterine distension which results in contractions and PPROM [4]. In our study the effect of maternal stress on preterm birth remained after exclusion of all the twins in the sample.

The relatively high AR1 might be due to our definition of maternal stress during pregnancy. We are not aware of any other study with this approach but if the study had concentrated on a specific diagnosis e.g., major depression the AR1 would probably have been lower. Clinical depression is suggested to carry up to a 2-fold increased risk for preterm delivery [16–17].

Our definition is interesting and important from a clinical perspective. It implies that even a stressor not causing a clinical diagnosis can have an impact on the risk for preterm birth. It seems that psychosocial risk factors e.g., financial problems, unemployment and unstable relationships can be of importance for the pregnancy outcome. A problem yet to be solved is what levels of stress affect the risk for preterm childbirth.

The findings in this study may also be the basis for discussions of the care of pregnant women exposed to stress. Future studies to examine interventions to reduce stress during pregnancy and the effect on preterm birth could be of importance.

## Conclusions

Preterm birth negatively affects the newborn child, both in the short and long term. In conclusion, approximately 20 % of the preterm births in this study were estimated to be due to maternal stress exposure during pregnancy as an attributable risk factor. Thus it is of great importance to identify and possibly alleviate the exposure to stress during pregnancy and by doing that try to decrease the preterm birth rate.

## Abbreviations

AOR: adjusted odds ratio
AR: attributable risk
IUFD: intrauterin fetal death
PPROM: preterm, premature rupture of membranes
SGA: small for gestation age
